# Early Developmental Responses to Seedling Environment Modulate Later Plasticity to Light Spectral Quality

**DOI:** 10.1371/journal.pone.0034121

**Published:** 2012-03-30

**Authors:** Eric J. B. von Wettberg, John R. Stinchcombe, Johanna Schmitt

**Affiliations:** 1 Biological Sciences, Florida International University, Miami, Florida, United States of America; 2 Center for Tropical Plant Conservation, Fairchild Tropical Botanic Garden, Coral Gables, Florida, United States of America; 3 Ecology and Evolution, University of Toronto, Toronto, Canada; 4 Ecology and Evolutionary Biology, Brown University, Providence, Rhode Island, United States of America; Instituto de Biología Molecular y Celular de Plantas, Spain

## Abstract

Correlations between developmentally plastic traits may constrain the joint evolution of traits. In plants, both seedling de-etiolation and shade avoidance elongation responses to crowding and foliage shade are mediated by partially overlapping developmental pathways, suggesting the possibility of pleiotropic constraints. To test for such constraints, we exposed inbred lines of *Impatiens capensis* to factorial combinations of leaf litter (which affects de-etiolation) and simulated foliage shade (which affects phytochrome-mediated shade avoidance). Increased elongation of hypocotyls caused by leaf litter phenotypically enhanced subsequent elongation of the first internode in response to low red∶far red (R∶FR). Trait expression was correlated across litter and shade conditions, suggesting that phenotypic effects of early plasticity on later plasticity may affect variation in elongation traits available to selection in different light environments.

## Introduction

Despite extensive research into the adaptive significance of phenotypic plasticity – the ability of genotype to produce multiple phenotypes under different conditions (e.g., [1]–[10])– far less work has examined the extent to which responses to multiple environmental cues interact to produce integrated phenotypes (e.g., [11]–[15]). Although plastic variation in traits in response to environmental cues has been documented in many plants (e.g., [16]–[22]), for most phenotypes, more than a single cue varies across suitable habitats and trait values in different conditions are integrated responses to these cues (*sensu* [12], [23]). Because of variation in multiple environmental cues, interactive effects are bound to occur on plastic phenotypes that respond to these cues [14], [24]–[27]. For responses to disparate cues that are driven by common molecular and hormonal pathways, there is a possibility for both synergistic and antagonistic interactions to occur between responses as, for example, may occur with jasmonic and salicylic acid-mediated plant defenses [28].

Plant growth is tightly regulated by light throughout the life cycle. Foliage shade reduces photosynethetically active radiation available to plants and is characterized by low ratios of red light (∼620–700 nm) to far red light (700–800), Many plants have the capacity for phenotypically plastic shade avoidance responses to foliage shade. A key component of shade avoidance is elongation, both as seedlings buried beneath leaf litter and soil, and as vegetative plants growing below an overhead plant canopy. In both the pre-photosynthetic seedling stage and photosynthetic vegetative stage, elongation in response to low R∶FR is controlled by the phytochrome family of photoreceptors, and involves the developmental responses of cell division and expansion [29]–[33] that may cause extensive correlations between responses at the two stages. When seedlings germinate under leaf litter or soil, they elongate their hypocotyls, the earliest emerging part of the stem, until they either reach appropriate light conditions for beginning autotrophic growth or run out of seed reserves. The cessation of elongation (de-etiolation) in response to light is partially mediated by phytochromes interacting with other photoreceptors and other signaling pathways [34]–[36].

After de-etiolation, plants will also elongate their stems during vegetative growth in response to shading by neighbors, which is sensed by reduced ratios of red to far-red (R∶FR) radiation reflected and refracted by foliage [35]. Because early height has a considerable impact on resource allocation and competitive success in plants [37], [38], and because both de-etiolation and shade avoidance elongation share common light receptors and downstream pathways, there is a high potential for physiological effects of early hypocotyl extension on subsequent shade avoidance. The relationships among traits involved in de-etiolation in different soil conditions could potentially constrain, enhance, or have no effect on subsequent shade avoidance. The nature of the effect will depend on whether trait expression has a genetic or an environmentally-induced basis, and on the nature of selection on various components of de-etiolation and shade avoidance.

One promising system to study the effects of delayed de-etiolation on shade avoidance responses is *Impatiens capensis*, a wetland annual native to eastern North America. *Impatiens capensis* has well characterized responses to shade (e.g., [17], [18], [27], [39]–[45]). Under low R∶FR its hypocotyls and internodes elongate [46]. When emerging from under leaf litter, *Impatiens* seedlings delay de-etiolation and elongate their hypocotyls more than they do when emerging from bare soil [47]. Leaf litter lowers R∶FR ratios at the soil surface [48]. It also creates a physical barrier at the soil surface, providing the soil below with some nutrients and potentially allelochemicals, and altering soil moisture and temperature. Both of these effects may favor elongation. Some woodland *Impatiens* genotypes respond to low R∶FR conditions generated by supplemental FR light localized to the first internode or first leaf with a suppression of elongation, rather than the elongation characteristic of the shade avoidance response by open habitat ecotypes [49]. This population-specific suppression of elongation, which is similar to the suppression of elongation that occurs in hypocotyl tissue during de-etiolation (the seedling high irradiance response, HIR), suggests that physiological responses of seedlings can have effects in later life that may vary among populations or genotypes.

Specifically, we asked the following questions: (1) Does early etiolation from under leaf litter affect subsequent elongation in response to foliage shade, and (2) is there genetic variation for this effect? We find that elongation through leaf litter enhances aspects of subsequent elongation, and that there is substantial genetic variation in responses to leaf litter and subsequent shade.

## Methods

### Study organism

*Impatiens capensis* Meerb. (Balsaminaceae) is an annual, self-compatible herb of North American deciduous forests and wetlands [50]. The species has a mixed mating system, commonly producing both outcrossing chamogamous flowers as well as self-fertilizing cleistogamous [51], allowing the production and maintenance of inbred lines. *I. capensis* occurs across a range of canopy habitats, and differentiated open and closed canopy forms have been observed (e.g., [17], [18], [27], [40], [41], [43], [44], [46], [52]).

### Line collection

To establish laboratory lines, seedlings were collected from natural populations in late April and early May of 2003 at the University of Connecticut, Storrs, CT and at Weetamo Woods, Tiverton, RI. No permits were required for this plant work. The species is common and not protected. Collections were made with the permission of the landowners (the University of Connecticut and the town of Tiverton, RI). Seedlings were excavated at two-meter intervals along ∼20 m by ∼20 m permanent grids in mixed woodland. Seedlings were transported to the Brown University greenhouse and grown in a common environment. Seeds from cleistogamous flowers were collected and stored in water in 96 well microtitre trays at 4°C for approximately four months [53] to establish inbred lines. Thirty inbred lines were used from each population.

### Experimental design

Our experimental design involved two litter treatments crossed with two shade treatments. We utilized plants from two populations with 30 lines per population, with five replicates per treatment combination (N = 1200 seeds planted, split evenly between two balanced blocks that corresponded to greenhouse benches). Seeds were planted on Dec 11, 2003 in the Brown University greenhouse. Seeds were either planted into bare potting soil (Metromix 360 corair) or planted into the same potting soil and then placed under 2 cm of leaf litter. The leaf litter was collected in a red oak forest adjacent to an *Impatiens* population in Medway, MA in July 2003 and stored at room temperature. Red oak (*Quercus rubra*) leaves are lobed and quite variable, but tend to be 12–20 cm in length and 5–10 cm in width. Ten-cm^2^ pots (0.52 l volume) were used, giving an experimental density of 100 plants per square meter, a common density in forest sites [45], [47], [53]. The assignment of litter treatment was at random within maternal family. Emergence was scored every other day until Jan 5, 2004. Final emergence and initial length of hypocotyls, first and second internodes, and initial total height were measured on Jan 5, 2004.

After scoring emergence and initial phenotypic traits, two shade treatments were established above the plants on Jan 6, 2004. Half of each block was assigned to a foliage shade (low R∶FR) treatment, and the other half to a neutral shade (equal R∶FR) treatment, both under natural light in the greenhouse. The foliage shade was created by affixing SRX-4 plastic sheets from Mitsui Corp (Japan) to PVC frames above the plants on the bench. The neutral shade was accomplished with clear plastic film. PAR was measured with a Decagon ceptometer, and R∶FR with a LI-COR spectroradiometer. Photosynthetically active radiation was 71% of full light under both shade treatments, equivalent to a light foliage shade. Full light at noon on Jan 4, 2004 was 154 micromoles/m/sec in the greenhouse, and 110 umol m−2 s−1 under the shade treatments. The R∶FR was 1.1 under the neutral treatment and 0.71 under the foliage shade treatment. These light treatments simulate the reduction in PAR that occurs as the forest canopy closes in Southern New England forests in spring, approximately one month after seedling germination. R∶FR reduction in these forests is limited until leaves on all trees have matured, about two months after germination. No supplemental lighting was used. Plants were harvested between Feb 10 and Feb 13, 2004. After harvest, we measured lengths of hypocotyl, the first two internodes, and total height, and recorded fruit production and final biomass. This lifespan is equivalent to that in woodland sites in New England where leaf litter and overhead foliage shade from the forest canopy is present ([18], Heschel et al unpublished]. In these conditions, total height tends to be much lower than in open sites [37]. We used fruit production, summed from fruits, immature fruits and flowers, and pedicels from early-produced fruits, as a fitness proxy, following [37], [40].

### Statistical analysis

#### Emergence

We sought to determine the phenotypic effect of leaf litter on subsequent shade avoidance responses. The effects of litter treatment on seedling emergence were examined with a chi-square test (Proc Freq, SAS v9.2, SAS Institute, Cary NC, USA).

#### Morphology before shade treatment

For initial measurements (before the imposition of the shade treatment), the design of our experiment was a randomized, blocked design, with litter treatment assigned at random to individuals. Thus for the pre-treatment measures, we used a mixed model ANOVA (Proc Mixed, SAS v9.2) in which traits (hypocotyl, first and second internodes, and height) were response variables, litter treatment and population were fixed effects, and block and genotype nested within population were random effects. We determined denominator degrees of freedom for F-tests of fixed effects with a Satterthwaite approximation, and test the significance of random effects with a likelihood ratio test. Specifically, the difference in −2 log-likelihoods in models with and without the genotype(population) term is χ^2^ distributed with 1 df.

#### Morphology after shade treatment

For traits collected at the final harvest, the design of our experiment was split block, with shade treatment being applied to sub-blocks, and leaf litter treatment applied at random within sub-blocks. Traits analyzed were hypocotyl length, internode lengths, total height, and total biomass. Litter, shade treatment, litter*shade, and population were included as fixed effects. Genotype nested within population, block, and the block by shade treatment interaction were random. The block*shade interaction was included to obtain proper F-tests for fixed effects. As before, we determined denominator degrees of freedom with Satterthwaite's approximations. For cases in which the block*shade variance component is estimated to be zero, it does not contribute to calculations of the degrees of freedom for F-tests of fixed effects. The significance of the genotype(population) term was calculated as described above. For cases where we detected statistically significant leaf litter treatment*foliage shade treatment interaction terms, we used least squared means contrasts to determine which of the treatment combinations differed.

Because of the complexity of our experimental design (some traits analyzed as random blocked design, some as split plot) and the different denominator degrees of freedom used by the Satterthwaite approximations (depending on whether block*shade variance components are greater than zero), it was not practical to implement MANOVA, We therefore analyzed each trait individually, correcting for multiple tests. using a false-discovery approach and estimated q-values for each P-value using the software Q-value [54]. Briefly, a q-value indicates the chance that a result is a false-positive, given that it is initially interpreted as significant [54]–[56]. The q-value approach has considerably more power than Bonferroni or other multiple testing procedures [54]. Our approach balances the need for proper F-statistics for each individual trait, while avoiding problems related to false-discovery. In general, the interpretation suggested by the q-values and p-values were concordant (i.e., statistically significant results had low chances of being false positives), and our results therefore focus primarily on traditional hypothesis testing (see below), although we present both *p* and *q* values.

In both our analyses of initial measurements (before shade treatment) and final measurements we considered population a fixed effect, as the two populations were chosen only because they produced sufficient seed in time for the start of the experiment, rather than considering them a random sample of the larger pool of *Impatiens* populations about which we wished to generalize. Likewise, we omitted population*litter and genotype(population)*litter interaction terms from statistical models, because our goal was not to test for population differentiation in litter plasticity. Genotype within population was considered random, as the number of lines and the spatial sampling of the collection (see [43]) were intended to be representative of within population variation.

A potential effect of emerging through leaf litter on subsequent shade avoidance could be an alteration of the expression of genetic variation. To determine whether there was equivalent variation in traits across leaf litter or simulated foliage shade treatments, we calculated both heritability and coefficients of genetic variation in all four environments, and used a bootstrapping approach to calculate standard errors around estimates [57]. All genetic parameters estimated in this study are broadsense parameters because inbred lines were used. Although they include non-additive effects, they are informative because most selection in highly selfing species like *Impatiens capensis* is by lineage sorting [58]. Although maternal effects are also included in this estimate, they are minimized by growing the parent of experimental plants under common garden conditions in the greenhouse.

#### Correlation of plasticities

We calculated plasticity to leaf litter and simulated foliage shade separately. To estimate plastic responses to leaf litter, we subtracted genotypic means under bare soil conditions from those under litter, separately for each R∶FR treatment; we used data on trait values measured before the imposition of the shade treatment. We calculated plasticity to R∶FR as the difference between the low and neutral R∶FR treatments, stratified by litter treatment, so that a genotype's plasticity to R∶FR is the difference in its mean value between foliage and neutral shade treatments within each litter treatment. We stratified the calculation of plasticities to R∶FR by litter treatment (and litter by R∶FR) so that we could examine the relationship between all plastic responses. To test for a relationship of plasticity to litter and overhead R∶FR, we calculated Pearson correlations of genotype mean plasticities (SAS Proc Corr). Calculations were performed separately for the two populations, as we observed differences in early growth, branching, and fitness between populations in our analysis above.

## Results

### Emergence

Emergence was lower under leaf litter (75%) than bare soil (84%; X2 df 1 = 15.5; p<0.0001). Emergence was lower in the CT population (69%) than in the RI population (90%, X2 df 1 = 82.2 p<0.0001) and differed among genotypes within both populations (X2 df 40 = 267.7, p<0.0001). Emergence did not take longer under leaf litter than bare soil (F_1, 880_ = 0.01, p = 0.92), suggesting that late emerging seedlings may have died without emerging from the litter.

### Morphology- before shade treatment

**Figure 1:**
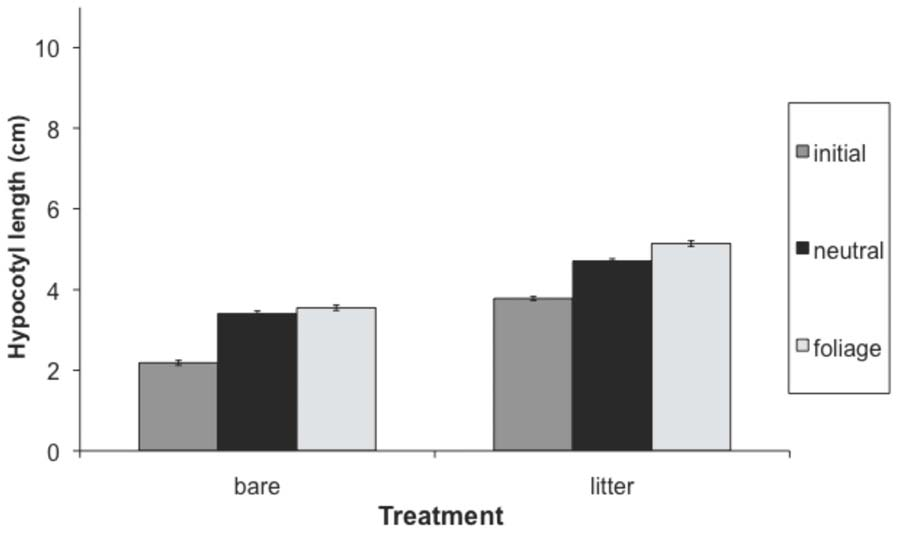
Effect of leaf litter and simulated foliage shade hypocotyl length. Values before and after the imposition of the shade treatment are shown. We present means across populations and treatments, with standard errors.

**Figure 2:**
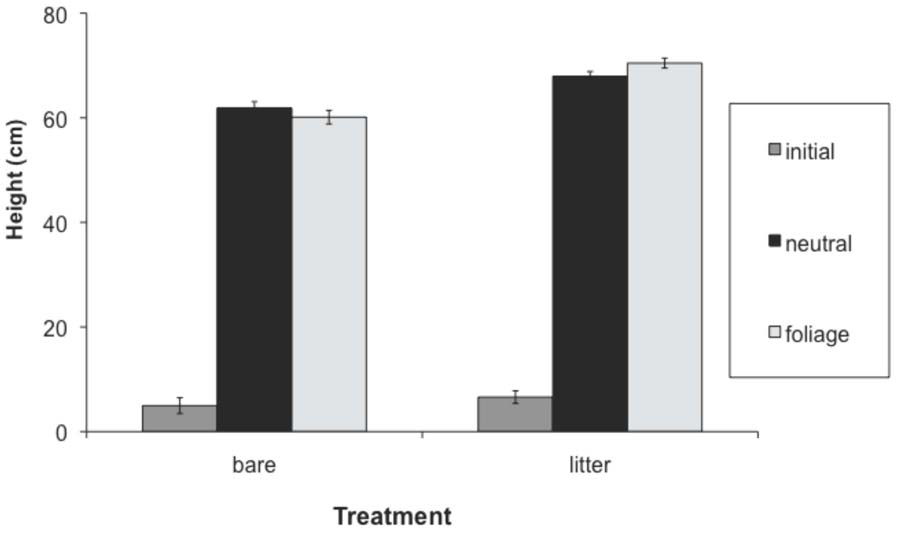
Effect of leaf litter and simulated foliage shade on first internode length. Values before and after the imposition of the shade treatment are shown. We present means across populations and treatments, with standard errors.

**Figure 3:**
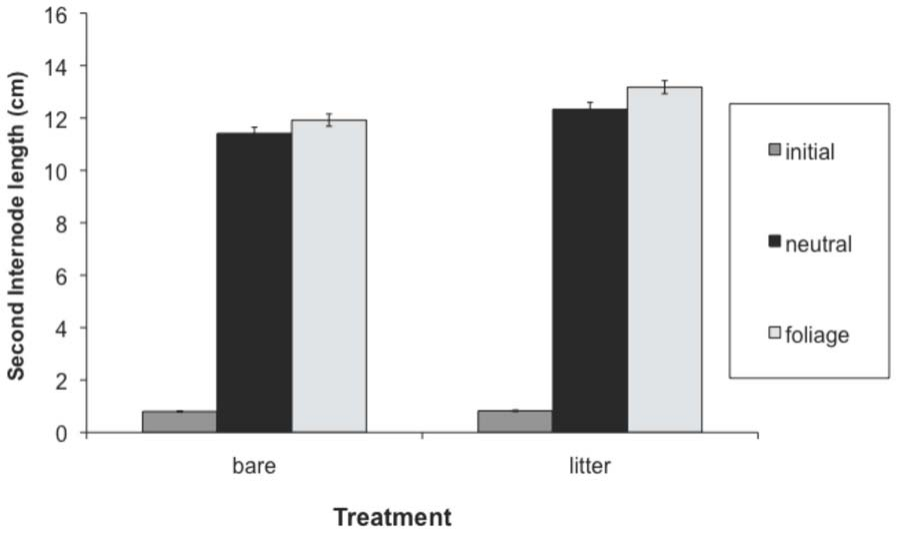
Effect of leaf litter and simulated foliage shade on second internode length. Values before and after the imposition of the shade treatment are shown. We present means across populations and treatments, with standard errors.

**Figure 4:**
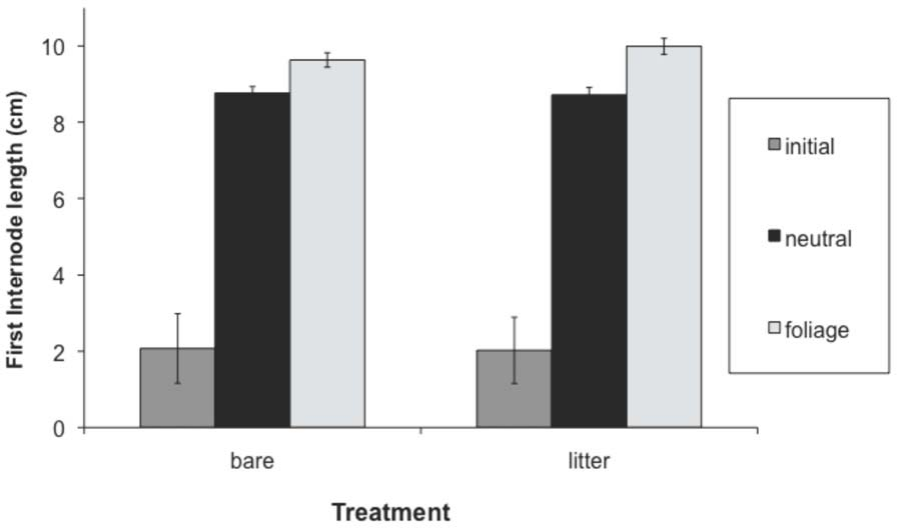
Effect of leaf litter and simulated foliage shade on total plant height. Values before and after the imposition of the shade treatment are shown. We present means across populations and treatments, with standard errors.

Hypocotyl length, first internode length and total height at the first census were greater in the presence than absence of leaf litter, while second internode length was not significantly affected by leaf litter (Table 1, Figs. 1, 2, 3, 4). Earlier emerging seedlings had longer hypocotyls (DF 1,880, F = 170, P<0.0001). The two populations differed significantly in all traits, with the RI population always being larger. We also observed significant variation among genotypes for all traits.

**Table 1:** Traits affected by leaf litter before the imposition of shade treatments.

	Hypocotyl Length	First Internode	Second Internode	Total Height
(A) Before Shade imposition				
Litter	**F_1,828_ = 1367.85,**	**F_1,801_ = 4.07,**	F_1,750_ = 2.87,	**F_1,826_ = 220.96,**
	**P<0.0001**	**P = 0.04**	P = 0.091	**P<0.0001**
	**Q = 0.0001**	**Q = 0.03**	**Q = 0.06**	**Q = 0.0001**
Pop	**F_1,52.6_ = 130.56,**	**F_1,52.6_ = 63.57,**	**F_1,53.2_ = 25.59,**	**F_1,82_ = 92.00,**
	**P<0.0001**	**P<0.0001**	**P<0.0001**	**P<0.0001**
	**Q = 0.0001**	**Q = 0.0001**	**Q = 0.0001**	**Q = 0.0001**
Genotype (population)	**χ^2^ = 66,**	**χ^2^ = 111.9,**	**χ^2^ = 71.4,**	**χ^2^ = 136.6,**
	**P<0.001**	**P<0.001**	**P<0.001**	**P<0.001**
	**Q = 0.0001**	**Q = 0.0001**	**Q = 0.0001**	**Q = 0.0001**

Leaf litter, R∶FR treatment, and population were fixed effects, with genotype nested within population a random effect. Only traits significantly affected by leaf litter or R∶FR manipulation are shown. Interaction terms with p>0.20 were removed from the models.

### Morphology- after shade treatment

Both leaf litter and simulated foliage shade induced elongation, and their effects were synergistic on hypocotyl length and total height (Table 2; Figs. 1, 2, 3, 4). Statistical decomposition of the significant leaf litter by simulated foliage shade interaction on hypocotyls by means contrasts showed that hypocotyls elongated more in response to simulated foliage shade when emerging from under leaf litter than from bare soil (estimated difference = 1.6411 cm, p<0.0001). Total height was increased by leaf litter and low R∶FR with an interaction between the two treatments (Table 2). The elongation effect of leaf litter was greater if followed by a low R∶FR treatment (Table 2, Fig. 4; estimated difference: 10.4515 cm, p<0.0001).

**Table 2:** Traits affected by leaf litter and R∶FR manipulation at the termination of the experiment.

	Hypocotyl length	First Internode	Second Internode	Total Height	Branches	Fitness
Litter	**F1,825 = 637.37,**	**F1, 825 = 1.66,**	**F1, 823 = 20.76,**	**F1, 825 = 46.40,**	F1, 824 = 7.44,	**F1, 826 = 23.95,**
	**P<0.0001**	**P = 0.20**	**P<0.0001**	**P<0.0001**	P<0.0001	**P<0.0001**
	**Q = 0.0001**	**Q = 0.12**	**Q = 0.0001**	**Q = 0.0001**	Q = 0.0001	**Q = 0.0001**
Shade	**F1,1 = 12.03,**	**F1, 824 = 40.68,**	**F1, 821 = 7.06,**	**F1, 824 = 0.05,**	F1, 1.98 = 0.00;	**F1, 2 = 0.20,**
	**P = 0.18**	**P<0.0001**	**P = 0.008**	**P = 0.82**	P = 0.95	**P = 0.70**
	**Q = 0.11**	**Q = 0.0001**	**Q = 0.006**	**Q = 0.40**	Q = 0.44	**Q = 0.35**
Litter *Shade	**F1, 819 = 6.15,**	**F1, 820 = 1.34,**	**F1,816 = 0.75,**	**F1, 819 = 6.09,**	F1, 819 = 0.19,	**F1, 822 = 0.02,**
	**P = 0.01**	**P = 0.25**	**P = 0.39**	**P = 0.014**	P = 0.67	**P = 0.89**
	**Q = .007**	**Q = 0.14**	**Q = 0.21**	**Q = 0.01**	Q = 0.34	**Q = 0.42**
Popul-ation	**F1, 50.2 = 1.2,**	**F1, 57.4 = 2.19,**	**F1, 51.9 = 0.34,**	**F1, 51.3 = 1.55,**	F1, 53.1 = 21.18,	**F1, 55.9 = 8.22,**
	**P = 0.28**	**P = 0.14**	**P = 0.56**	**P = 0.22**	P<0.0001	**P = 0.0058**
	**Q = 0.16**	**Q = 0.09**	**Q = 0.30**	**Q = 0.13**	Q = 0.0001	**Q< = 005**
Geno	**χ2 = 51,**	**χ2 = 133.1,**	**χ2 = 61.4,**	**χ2 = 60.8,**	χ2 = 115.3,	**χ2 = 142.2,**
(Pop)	**P<0.0001**	**P<0.0001**	**P<0.0001**	**P<0.0001**	P<0.0001	**P<0.0001**
	**Q = 0.0001**	**Q = 0.0001**	**Q = 0.0001**	**Q = 0.0001**	Q = 0.0001	**Q = 0.0001**

Leaf litter, R∶FR treatment, and population were fixed effects, with genotype nested within population a random effect. Only traits significantly affected by leaf litter or R∶FR manipulation are shown. Interaction terms with p>0.20 were removed from the models.

After the imposition of shade treatment, both first and second internode length were significantly increased by low R∶FR (Table 2, Fig. 2, 3). Second internode length was also affected by litter treatment (Table 2, Fig. 3) after the imposition of the shade treatments. Branching was greater in the presence of leaf litter, particularly in the CT population, which had significantly more branching than the RI population (Table 2). Fitness, estimated as total reproduction from the sum of all fruits produced, was significantly higher in the presence of leaf litter (Table 2), but did not differ significantly between shade treatments. There was significant variation among genotypes for all traits. Estimates of broad sense heritability and coefficients of genetic variation across traits and populations support the conclusion that significant genetic variation exists for elongation traits (Tables S1, S2).

### Correlations of plasticities

There was little evidence for a within-population correlation between genotypic-mean plasticity to leaf litter and subsequent plasticity to simulated foliage shade in either population (Tables 3, 4). There was no correlation of responses to leaf litter and responses to foliage shade for plants grown on bare soil (Tables 3, 4). In both populations there was a weak correlation of the plasticity of the first internode to leaf litter and the subsequent plasticity of the first and second internodes and total height to simulated foliage shade. This weak correlation was only significant if subsequent plasticity to foliage shade was calculated in plants that received the leaf litter treatment; it was not significant in plants from the bare soil treatment (Tables 3, 4). Genotype mean plasticities of traits to simulated shade were not significantly positively correlated across leaf litter treatments for any traits examined in either population (Table 5).

**Table 3:** Correlation of genotype mean plasticity to leaf litter and simulated foliage shade for the CT population.

Plasticity (difference between treatments) of traits	Hypocotyl (leaf litter plasticity)	First Internode (leaf litter plasticity)	Second Internode (leaf litter plasticity)	Total Height (leaf litter plasticity)
Hypocotyl (R∶FR plasticity, under Bare soil)	−0.16	0.10	0.17	0.03
First internode (R∶FR plasticity, under Bare soil)	−0.26	0.28	0.01	−0.15
Second internode (R∶FR plasticity, under Bare soil)	−0.17	−0.22	−0.15	−0.33
Total height (R∶FR plasticity, under Bare soil)	−0.03	0.13	−0.16	−0.08
Hypocotyl (R∶FR plasticity, under leaf litter)	0.13	0.13	−0.08	0.07
First internode (R∶FR plasticity, under leaf litter)	0.13	0.31	−0.21	0.07
Second internode (R∶FR plasticity, under leaf litter)	0.07	0.53**	0.01	0.27
Total height (R∶FR plasticity, under leaf litter)	0.026	0.45*	−0.05	0.31

Plasticity to leaf litter was calculated from measurements made before the imposition of the shade treatment. Plasticity to foliage shade was calculated separately for plants in the bare soil and leaf litter treatments. r values are shown, with * for p<0.05, ** for p<0.01.

**Table 4:** Correlation of genotype mean plasticity to leaf litter and simulated foliage shade for the RI population.

Plasticity (difference between treatments) of traits	Hypocotyl (leaf litter plasticity)	First Internode (leaf litter plasticity)	Second Internode (leaf litter plasticity)	Total Height (leaf litter plasticity)
Hypocotyl (R∶FR plasticity, under Bare soil)	−0.01	0.10	−0.27	−0.10
First internode (R∶FR plasticity, under Bare soil)	−0.09	−0.23	−0.12	−0.22
Second internode (R∶FR plasticity, under Bare soil)	−0.09	0.07	0.11	0.12
Total height (R∶FR plasticity, under Bare soil)	0.002	0.08	0.29	0.22
Hypocotyl (R∶FR plasticity, under leaf litter)	−0.06	0.30	0.05	0.03
First internode (R∶FR plasticity, under leaf litter)	−0.01	0.39*	0.08	0.25
Second internode (R∶FR plasticity, under leaf litter)	0.17	0.49**	0.12	0.32
Total height (R∶FR plasticity, under leaf litter)	0.20	0.16	−0.14	0.14

Plasticity to leaf litter was calculated from measurements made before the imposition of the shade treatment. Plasticity to foliage shade was calculated separately for plants in the bare soil and leaf litter treatments. r values are shown, with * for p<0.05, ** for p<0.01.

**Table 5:** Correlation between genotypic mean plasticity to simulated foliage shade in the bare soil and leaf litter treatments.

Traits	Correlation, CT population	Correlation, RI population
Hypocotyl	−0.07	0.14
First Internode	0.24	−0.22
Second Internode	0.004	0.11
Total Height	0.12	0.03

Calculations were performed separately for two populations, with r-values for the CT and RI shown respectively. No correlation was significant at p<0.05.

## Discussion

We found little evidence that elongation traits in *Impatiens* are constrained by physiological or pleiotropic correlations with de-etiolation expressed in response to leaf litter. Rather, delayed de-etiolation and elongation of hypocotyls caused by emerging from under leaf litter phenotypically enhanced subsequent elongation of the first internode in response to simulated foliage shade. Our results suggest that leaf litter will differentially affect the ability of ecotypes to respond to foliage shade, and that variation in the relationship of these two responses may exist in *Impatiens*.

### How leaf litter affects subsequent elongation responses to foliage shade

Leaf litter has several consequences that could affect subsequent responses to foliage shade. These effects include lowering R∶FR ratios below leaf litter [48], lowering total light levels, creating a physical barrier at the soil surface, providing the soil below with some nutrients and potentially allelochemicals, and altering soil moisture and temperature. In this experiment we watered and fertilized plants so that these resources were not limiting. We have collected soil temperature data from *Impatiens* populations across Rhode Island in early spring, and found that forest understory (high litter) and open canopy (low litter) sites do not differ markedly in temperature at the soil surface, suggesting that the temperature effect is not large (E.v.W. unpublished data). Hypocotyl length can also be affected by other factors such as negative gravitropism, phototropism and thigmorphogensis (e.g., [27]). Increasing total available light can promote de-etiolation but ultimately increase total plant height in *Impatiens* [46], and has the same effect on seedlings ([59], von Wettberg, Stinchcombe and Schmitt, personal observation].

Plants growing in leaf litter have to extend their shoots more to penetrate the leaf litter and reach the light than plants growing under bare soil, which might select for greater hypocotyls elongation under both neutral and foliage shade conditions [47]. Plants emerging from leaf litter may have become more elongated in foliage shade, as observed in this experiment, because of a priming effect, whereby plants already elongated in response to leaf litter are more able to elongate in response to shade. Because seedlings frequently have to find their way through leaf litter, seedlings emerging from leaf litter can grow laterally across the soil surface a few centimeters from where they germinated before growing vertically from the soil surface. To support an upright plant, the seedling must straighten its hypocotyls and first internode, possibly by structurally reinforcing the stem (see [27]). This process could lead to plants that are initially larger than neighbors in bare soil, leading to an initial head start or asymmetric size advantage [37], [60] that contributes to the higher fitness we observed here for plants grown in leaf litter. Plants that responded to leaf litter with an elongation response were taller at the time the shade treatment was imposed, despite having had to expend energy penetrating the leaf litter, and were more able to respond to the shading signal.

### Are de-etiolation and shade avoidance elongation correlated in *Impatiens*?

De-etiolation and shade avoidance may be partially correlated because they are both elongation responses of stem tissue that may be at least partially mediated through phytochrome photoreceptors. Although there is a mechanistic basis for this correlation, it could take several forms. De-etiolation can have a purely phenotypic effect on subsequent shade avoidance, by either limiting or enhancing it. Secondly, if there is genetic variation in either response, it is possible that there is covariation between the plasticities.

We found little evidence for a correlation between genotype mean plasticities to different soil conditions and shade conditions. Accordingly, although responses to soil surface leaf litter and shading may share at least one overlapping set of photoreceptors (phytochromes), they do not strongly constrain each other under the conditions examined here. Seedling and adult phenotype plants are of course affected by multiple forces in addition to light quality, such as irradiance, temperature, negative gravitropism, positive phototropism, and thigmotropism, which can also interact with each other [27]. We also find little evidence of a negative correlation between plasticity to leaf litter and foliage shade, suggesting that although trade-offs may exist between early and late elongation, they are either not strong or our experimental conditions did not uncover them. Instead, we observed a purely phenotypic effect, with elongation in response to leaf litter enhancing subsequent shade avoidance for all genotypes.

The two other experiments that have looked at correlations between phyotchrome-mediated plasticities early and later in plant development have either observed only a phenotypic effect of early elongation on late elongation [61] or have found no evidence of a genetic correlation between phytochrome-mediated plasticies [62]. Weinig and Delph [61] found that early elongation in response to low R∶FR lowered the phenotypic response of *Abutulon theophrasti* plants to subsequent low R∶FR when compared to the responses of plants exposed to neutral shading, but did not report genotypic correlations that may have underlain this phenotypic effect. Under variable field conditions, Weinig [19] found that the phenotypic relationship between early and late internode elongation in *A. theophrasti* changed from positive to negative depending on whether shading from competitors occurred primarily early or late in the season, and depending on the ability of *A. theophrasti* plants to overtop these competitors. Although populations differ in their shade avoidance responses [19], that study found no evidence of a positive or negative genetic correlation between early and late shade avoidance elongation. Botto and Smith [62] tested for a genetic correlation between hypocotyl elongation and accelerated flowering time under low R∶FR in over 100 *Arabidopsis thaliana* ecotypes, and found no evidence of a genetic correlation between these phytochrome-mediated plasticities.

### Will leaf litter affect the evolution of shade avoidance?

Both leaf litter and foliage shade have been shown to have fitness impacts when examined alone in *Impatiens* [17], [18], [39], [40], [47], [58]. But how do the two affect each other? Our results concur with similar evidence from other species showing only a phenotypic correlation between developmentally early and late phytochrome-mediated plasticities [61], [62], although the lack of strong correlations may simply result from a limitation of experimental conditions. Genotype mean plasticities to leaf litter and foliage shade were largely uncorrelated, with the exception of weak correlations for first internode and total height. We conclude that de-etiolation and shade avoidance responses can evolve quite independently. Although phytochrome-mediated shade avoidance clearly varies between many species (e.g., [63], [64]) and populations within species (e.g., [20], [65], [66]), including *Impatiens* (e.g., [41], [43]), joint evolution of shade avoidance elongation with other phytochrome-mediated plasticities does not appear to occur.

## Supporting Information

Table S1 Broad sense heritability between leaf litter and shade treatments. Standard deviations are calculated from 10,000 bootstraps.
(DOCX)

Table S2 Coefficient of genetic variation between leaf litter and shade treatments. Standard deviations are calculated from 10,000 bootstraps.
(DOCX)
