# Supplementary material for: Early Developmental Responses to Seedling Environment Modulate Later Plasticity to Light Spectral Quality
Source: PLoS One. 2012 Mar 30;7(3):e34121. doi: 10.1371/journal.pone.0034121 (PMC3316606; doi:10.1371/journal.pone.0034121)
Supplement: Table S1 — Broad sense heritability between leaf litter and shade treatments. Standard deviations are calculated from 10,000 bootstraps. (DOCX) [file pone.0034121.s001.docx]

Table S1. Broad sense heritability between leaf litter and shade treatments. Standard deviations are calculated from 10,000 bootstraps.

|  | CT | CT | CT | CT |  | RI | RI | RI | RI |
| --- | --- | --- | --- | --- | --- | --- | --- | --- | --- |
| Trait | Bare Foliage | Bare Neutral | Leaf Foliage | Leaf Neutral |  | Bare Foliage | Bare Neutral | Leaf Foliage | Leaf Neutral |
| Hypocotyl | 0.40  *+/- 0.05* | 0.46  *+/- 0.07* | 0.14  *+/- 0.06* | 0.30  *+/- 0.04* |  | 0.28  *+/- 0.05* | 0.33  *+/- 0.06* | 0.24  *+/- 0.05* | 0.43  *+/- 0.06* |
| First Internode | 0.28  *+/- 0.06* | 0.55  *+/- 0.10* | 0.21  *+/-0.05* | 0.24  *+/- 0.06* |  | 0.33  *+/- 0.11* | 0.49  *+/- 0.07* | 0.34  *+/- 0.03* | 0.55  *+/- 0.07* |
| Second Internode | 0.33  *+/- 0.05* | 0.40  *+/- 0.08* | 0.16  *+/- 0.05* | 0.36  *+/- 0.04* |  | 0.19  *+/- 0.06* | 0.45  *+/- 0.06* | 0.39  *+/-0.06* | 0.69  *+/-0.16* |
| Height | 0.32  *+/- 0.05* | 0.36  *+/- 0.06* | 0.35  *+/- 0.06* | 0.32  *+/- 0.06* |  | 0.28  *+/- 0.04* | 0.41  *+/- 0.05* | 0.34  *+/- 0.04* | 0.41  *+/-0.06* |
