# Supplementary material for: Early Developmental Responses to Seedling Environment Modulate Later Plasticity to Light Spectral Quality
Source: PLoS One. 2012 Mar 30;7(3):e34121. doi: 10.1371/journal.pone.0034121 (PMC3316606; doi:10.1371/journal.pone.0034121)
Supplement: Table S2 — Coefficient of genetic variation between leaf litter and shade treatments. Standard deviations are calculated from 10,000 bootstraps. (DOCX) [file pone.0034121.s002.docx]

Table S2. Coefficient of genetic variation between leaf litter and shade treatments. Standard deviations are calculated from 10,000 bootstraps.

| Population | CT | CT | CT | CT |  | RI | RI | RI | RI |
| --- | --- | --- | --- | --- | --- | --- | --- | --- | --- |
| Trait | Bare Foliage | Bare Neutral | Leaf Foliage | Leaf Neutral |  | Bare Foliage | Bare Neutral | Leaf Foliage | Leaf Neutral |
| Hypocotyl | 0.58,  *+/- 0.03* | 0.79  *+/- 0.1* | 0.39  *+/- 0.03* | 0.43  +/- 0*.03* |  | 0.48  +/- 0*.03* | 0.38  +/- 0*.04* | 0.53  +/-*0.04* | 0.53  +/- 0*.05* |
| First Internode | 1.7  +/- 0*.1* | 3.8  +/- 0*.9* | 1.2  +/- 0*.2* | 1.4  +/- 0*.1* |  | 2.2  +/- 0*.2* | 1.6  +/- 0*.2* | 1.4  +/- 0*.1* | 1.7  +/-0*.2* |
| Second Internode | 2.5  +/- *0.2* | 2.8  +/- 0.1 | 1.4  +/- 0.2 | 2.4  +/- 0.2 |  | 1.0  +/- 0.1 | 1.7  +/- 0.1 | 2.5  +/- *0.2* | 3.7  +/- *0.9* |
| Height | 10.7  +/- *0.8* | 9.7  +/- *0.7* | 7.5  +/- *0.5* | 6.4  +/- *0.8* |  | 8.2  +/- *0.7* | 7.2  +/- *0.5* | 7.5  +/- *0.5* | 8.0  +/- *0.4* |
